# Mechanisms of Action of Brief Alcohol Interventions Remain Largely Unknown – A Narrative Review

**DOI:** 10.3389/fpsyt.2014.00108

**Published:** 2014-08-26

**Authors:** Jacques Gaume, Jim McCambridge, Nicolas Bertholet, Jean-Bernard Daeppen

**Affiliations:** ^1^Alcohol Treatment Center, Department of Community Health and Medicine, Lausanne University Hospital, Lausanne, Switzerland; ^2^Department of Social and Environmental Health Research, Faculty of Public Health and Policy, London School of Hygiene & Tropical Medicine, London, UK

**Keywords:** brief intervention, alcohol, mechanisms, active ingredients, components, mediators, motivational interviewing

## Abstract

A growing body of evidence has shown the efficacy of brief intervention (BI) for hazardous and harmful alcohol use in primary health care settings. Evidence for efficacy in other settings and effectiveness when implemented at larger scale are disappointing. Indeed, BI comprises varying content; exploring BI content and mechanisms of action may be a promising way to enhance efficacy and effectiveness. Medline and PsychInfo, as well as references of retrieved publications were searched for original research or review on active ingredients (components or mechanisms) of face-to-face BIs [and its subtypes, including brief advice and brief motivational interviewing (BMI)] for alcohol. Overall, BI active ingredients have been scarcely investigated, almost only within BMI, and mostly among patients in the emergency room, young adults, and US college students. This body of research has shown that personalized feedback may be an effective component; specific MI techniques showed mixed findings; decisional balance findings tended to suggest a potential detrimental effect; while change plan exercises, advice to reduce or stop drinking, presenting alternative change options, and moderation strategies are promising but need further study. Client change talk is a potential mediator of BMI effects; change in norm perceptions and enhanced discrepancy between current behavior and broader life goals and values have received preliminary support; readiness to change was only partially supported as a mediator; while enhanced awareness of drinking, perceived risks/benefits of alcohol use, alcohol treatment seeking, and self-efficacy were seldom studied and have as yet found no significant support as such. Research is obviously limited and has provided no clear and consistent evidence on the mechanisms of alcohol BI. How BI achieves the effects seen in randomized trials remains mostly unknown and should be investigated to inform the development of more effective interventions.

## Introduction

A growing body of evidence has shown the efficacy of brief intervention (BI) for hazardous alcohol use in primary health care settings (1). In a review of systematic reviews and meta-analyses of the effects of alcohol BI in primary health care, O’Donnell and colleagues (1) found 34 systematic reviews covering a total of 56 randomized controlled trials reporting about 80 papers, among which it was consistently reported that BI was efficacious for addressing hazardous and harmful drinking in primary health care, particularly in middle-aged, male drinkers. However, even within this important body of research, it was limited on the effects of BI among certain groups such as women, older and younger drinkers, minority ethnic groups, dependent and other co-morbid drinkers, and those living in transitional and developing countries (1). They also concluded that evidence was lacking as regards to the optimum length and frequency of BI, as well as the optimum content of BI. Furthermore, recent null findings from large pragmatic trials (2, 3) have called into question the extent to which the systematic review evidence on BI efficacy can be generalized to effectiveness in routine primary care, and further pointed to the lack of knowledge on intervention content and active ingredients.

As primary health care providers have the ability to reach a broad population, primary health care was identified as the most desirable setting within the health care system to screen, identify, and deliver BI to people with hazardous or harmful drinking. As such, most of the BI research from 1980s onward was designed and conducted in primary health care [even though seminal studies of BI were originally conducted in the emergency department, see Ref. (4)]. This may explain why primary health care is the setting in which evidence of BI efficacy has been most established. However, BI has been implemented and tested in several other settings such as general hospitals (5), emergency departments (6), and colleges and universities (7, 8). While BI has been shown to produce small effects within US college settings (7, 8), evidence has been surprisingly slow to accumulate in other settings (9), and additional research is required to investigate mixed findings, refine current practice guidelines, and continue to bridge the gap between science and practice (10).

Brief intervention is an umbrella term that is used to describe a quite heterogeneous group of interventions, from advice to more personalized forms of intervention based on motivational interviewing (MI). This heterogeneity in intervention content may well explain some of the inconsistencies observed in intervention effects across studies. Differences in setting characteristics (e.g., ongoing vs. single contacts with health care provider, professional training, and context of delivery) may also explain differences in efficacy.

In many ways, BI research has been conducted as if the intervention could be treated as a black box, without regard for detailed content, as has been the case for most behavioral treatments (11). The problem is that, over the years and across studies, the black box content has been drastically modified (12), with little, if any, careful study of the implications. Researchers have not deployed the same diligence in efforts to study BI content as has been done for the study of the efficacy of the different versions of the black box.

Conflicting evidence between efficacy studies and pragmatic trials, as well as between studies conducted in different contexts and settings might be explained by the wide range of interventions, and the effects of setting characteristics, on the various hypothesized active ingredients of efficacy. For these reasons, it has been suggested that “BI content matters” in research (13) is of great importance to identify which element of intervention may be related to efficacy, in order to develop more effective interventions. It is also crucial for implementation since training clinicians to deliver BIs is challenging, particularly so when key skills needed for the accomplishment of key tasks remain to be clarified. Therefore, in order to establish the state of current knowledge about which elements of content matter, we conducted a review of studies that reported on mechanisms of action of BI for hazardous or harmful alcohol use. This is fundamentally a hypothesis generation study, seeking to identify important targets for further study.

## Materials and Methods

### Inclusion criteria

We included publications meeting the following criteria: (1) the intervention was described as “BI,” “brief advice,” “brief motivational intervention,” or “BMI”; (2) the intervention targeted alcohol; (3) the intervention was delivered face-to-face (i.e., group interventions and computer interventions were excluded); (4) some mechanism of intervention effect was investigated; and (5) the publication was either an original research article or a literature review, published in a peer-reviewed journal. Literature reviews were included if at least part of the content met the above criteria (i.e., reviews comparing different type of interventions were included if some but not all studied interventions met inclusion criteria).

### Data collection

The electronic databases PubMed and PsychInfo were first searched for studies meeting the aforementioned inclusion criteria. We had three key constructs, which were operationalized for keywords searches as follows: active ingredient (component, mechanism, or process); BI (brief advice, brief motivational intervention, or BMI); and alcohol (drinking). Then, we reviewed references of retrieved publications.

### Data analysis

Retrieved articles that met inclusion criteria were very heterogeneous with respect to their type, methods, and focus. It was apparent that meta-analysis would not be appropriate or feasible. We thus chose to analyze the retrieved articles in topics and types of mechanisms, and to present them in a narrative review format. The key distinction in the included evidence-base pertains to two different types of mechanisms: BI components (i.e., intervention strategies, or components, that were isolated and analyzed as possible predictors of enhanced effects), and BI effect mediators (i.e., psychological dimensions, psycholinguistic behaviors, or cognitive states affected by the intervention and associated with targeted behavior change). A short introduction and discussion of evidence for each mechanism is presented below. The discussion at the end of the paper offers a more general synthesis and overview of possible implications for further developments in BI research.

## Results

### Components

In their early review of BI for alcohol problems (which included both opportunistic BI for non-treatment seekers, where the research comparison is with no or more minimal intervention, and BI for treatment seekers, where the comparison is with longer forms of regular treatment), Bien and colleagues (14) showed that BI (a) were usually significantly more effective than no intervention, (b) commonly showed similar impact to that of more extensive interventions, and (c) could enhance the effectiveness of subsequent treatment. In the second part of this article, they reviewed common elements of effective BIs, and six elements summarized by the acronym FRAMES (feedback, responsibility, advice, menu, empathy, and self-efficacy) were identified for further study.

In the review of systematic reviews of alcohol BI studies in primary health care (1), the authors found few reviews considering the impact of the actual content of interventions on their effectiveness (15–18). In general, these reviews highlighted the lack of available evidence on this issue, mainly due to the heterogeneity of the included studies (1). Whitlock and colleagues (17) reported that all interventions demonstrating statistically significant improvements in alcohol outcomes included at least two of the three key elements: feedback, advice, and goal setting. Different BI components highlighted in the empirical studies of BI mechanisms or derived from meta-analyses are presented below.

#### Feedback

Early BI models have focused explicitly on feedback of risk or harm as a tool for instigating change (14). In the review on alcohol BI in primary health care by Bertholet and colleagues (19), all BI models but one included feedback. The role of feedback within BI has more recently been empirically questioned in the studies reported below.

Murphy and colleagues (20) evaluated the relative efficacy of personalized drinking feedback delivered with and without BMI among 54 drinking college students. At 6-month follow-up, participants in both groups showed significant, small to moderate reductions in alcohol consumption, but the groups did not differ. The hypothesis that a BMI would enhance the efficacy of feedback was thus not supported. Another study (21) evaluated the relative efficacy of BMI and feedback among 122 hazardous drinking college students. Participants were randomized to (a) BMI with feedback, (b) BMI without feedback, (c) mailed feedback only, (d) BMI with mailed feedback, or (e) assessment-only control. At 2-month follow-up, all groups reduced their consumption, peak BAC, consequences, and dependence symptoms, with no significant difference between groups. Walters and colleagues (22) used a similar design among 279 heavy-drinking students, which were randomized to (a) web feedback only, (b) a single BMI session without feedback, (c) a single BMI session with feedback, or (d) assessment only. At 6-month follow-up, BMI with feedback significantly reduced drinking, as compared with assessment only (effect size = 0.54), BMI without feedback (effect size = 0.63), and feedback alone (effect size = 0.48). Neither BMI alone nor feedback alone differed from assessment only.

One study (23) evaluated the costs and cost-effectiveness of combining BMI with feedback to address heavy drinking among university freshmen (i.e., first year), with a total of 727 students randomized to four conditions: (a) assessment only, (b) BMI only, (c) feedback only, and (d) BMI with feedback, followed-up 3 months later. Cost–effectiveness analyses showed that despite being the most expensive intervention, BMI with feedback was the most effective intervention and might be a cost–effective intervention.

In their meta-analysis of prevention interventions for drinking college students, Carey and colleagues (8) suggested that individual, face-to-face interventions using MI and personalized normative feedback predicted greater reductions in alcohol-related problems than other interventions. In their subsequent meta-analysis (7), face-to-face interventions including feedback were significantly more effective on alcohol outcomes than interventions not including it.

Brought together, the studies presented above suggest that feedback might be an important component of BMI, but some caveats should be noted. Meta-analytic findings were supportive of the use of feedback (7, 8, 14). These are, however, observational data, and other study characteristics may be relevant. Studies that experimentally investigated this question via dismantling the relative efficacy of feedback and BMI produced more mixed findings. Two studies showed significantly enhanced effects when BMI included feedback (22, 23), while two other found equivalent effects (20, 21), thus showing no impact of feedback. It must, however, be noted that the latter two studies had smaller sample sizes. An important limitation to these findings is that, with the exception of the meta-analysis by Bien and colleagues (14), all studies reported above included only US college students.

#### Decisional balance

The decisional balance is a brief detailing of the advantages (the “pros”) and disadvantages (the “cons”) of behavior change, originally conceptualized by Janis and Mann (24), which has become a critical construct in the transtheoretical model of behavior change (25) and a common component of BI (or at least BMI).

Three studies empirically evaluated the effects of decisional balance as a stand-alone BI, or as a component of alcohol BI (26–28), all within the US college setting. Collins and Carey (26) examined the effects of decisional balance exercises on measures of risky drinking among college students with alcohol-related problems (*N* = 131). Students were randomized to (a) an in-person 30-min decisional balance discussion, (b) a written decisional balance, or (c) an assessment-only control group. No significant differences among the groups were found at 2-week and 6-month follow-up on alcohol consumption, heavy-drinking episodes, alcohol consumption during peak drinking occasions, and alcohol-related problems. In another randomized controlled trial (27), the authors compared (a) a basic BMI, (b) BMI enhanced with a decisional balance module, and (c) an assessment-only control group. Assessments at 1, 6, and 12 months showed that the basic BMI improved all drinking outcomes beyond the effects of the assessment-only control group at 1 month, whereas the enhanced BMI did not. Risk reduction achieved by both BMI models maintained throughout the follow-up year. Thus, both studies did not provide support for decisional balance as an effective component of BI or stand-alone BI for at-risk drinking college students.

LaBrie and colleagues (28) examined the impact of decisional balance among 47 men in the college setting. The students completed questionnaires on alcohol use and unsafe sexual practices and were engaged in a discussion of pros and cons of decreasing their drinking, but not of safer sex. One-month follow-up data showed statistically significant decreases in drinking, but no change in sexual behaviors. This study thus suggests a potential impact of decisional balance, but the small size and design of the study limits confidence in its conclusions.

Two meta-analyses also tested whether interventions including decisional balance were more effective than interventions not including it. In a meta-analysis of 62 controlled studies evaluating prevention interventions for drinking college students (8), it was suggested that the interventions were somewhat more successful at reducing alcohol-related problems at short-term follow-up if the intervention content contained a decisional balance exercise (*B* = 0.17, *p* = 0.05). However, in their more recent meta-analytic review of BI for college students, Carey and colleagues (7) found that the reductions in quantity of alcohol consumption (per week/month) were smaller when face-to-face BIs included a decisional balance exercise (*B* = -0.60, *p* = 0.04, 7 studies including decisional balance compared to 26).

The abovementioned analyses showed mixed findings, and tend to suggest a potential detrimental effect of the decisional balance exercise. Miller and Rose (29) have suggested that decisional balance may be both theoretically and empirically contraindicated with ambivalent people when the goal of treatment is to foster change. They recommended that clinicians using MI to help clients resolve ambivalence and to promote behavior change should not include decisional balance as a part of the intervention. For these authors, evocation of change talk (i.e., only one part of the decisional balance) is more appropriate when the clinician intends to help clients resolve ambivalence in the direction of change.

#### MI skills

Among the essential effective BI components summarized by the FRAMES acronym (14), several are directly shared with MI (30). This is the case for the emphasis on personal responsibility for change (i.e., patients are advised that change in drinking is their own responsibility and choice), therapeutic empathy as a counseling style (i.e., warm, reflective, and understanding approach in opposition to directive, aggressive, authoritarian, or coercive elements), and enhancement of client’s self-efficacy for change (i.e., optimism regarding the possibility of change rather than emphasizing helplessness or powerlessness). In the meta-analysis on prevention interventions for drinking college students, Carey and colleagues (8) showed that interventions using MI predicted greater reductions in alcohol-related problems.

Several studies did directly and empirically addressed MI skills as active ingredients of alcohol BI and are presented below. McNally and colleagues (31) examined the role of five MI components in a BMI for heavy episodic alcohol use among college students (*N* not specified, random half of 73 participants included in the study). These components were evaluated by the students at post-intervention. Two of these were MI skills (perceived empathy and relative focus on personal responsibility for change). Partial correlations were conducted between the individual component and a composite alcohol involvement score measured at 6-week follow-up (controlling for baseline drinking). Participants’ subjective experience of the relative focus on personal responsibility for change was not significantly associated with outcome in these analyses. However, findings suggested that BMI participants who reported a greater sense of perceived empathy from the counselor were more likely to show lower levels of alcohol involvement at follow-up.

Feldstein and Forcehimes (32) examined the specific role of empathy in a BMI for alcohol use among underage heavy-drinking college students. Contrary to predictions, empathy was not correlated with 2-month outcomes (binge drinking and alcohol-related problems). Authors noted, however, that limited variability existed for empathy, due to therapists’ consistent high performance on the empathy variable (mean of 6.92, SD = 0.27 on a scale of 1–7) and that the sample was small (*N* = 35).

Gaume and colleagues (33) tested several counselors’ behaviors as predictors of change in alcohol use among patients in the emergency department receiving BMI. Counselor’s empathy was correlated with decreases in alcohol use (baseline to 12-month follow-up difference) but this association was no longer significant when a significant patient predictor (patient ability to change, see below) was covaried. Using the same data, however, these authors used multilevel models to test MI skills taking clustering within counselors into account (34). Findings showed that counselors with better MI skills achieved better outcomes overall and maintained efficacy across all levels of the significant patient predictor mentioned above (i.e., patient ability to change). On the other hand, counselors with poorer MI skills were effective mostly at high levels of ability to change. Findings indicated that avoidance of MI-inconsistent skills was more important than frequency of using MI-consistent skills and that training and selection of counselors should be based more on an overall MI-consistent attitude (combining acceptance, MI spirit, confrontation and warning avoidance, use of complex reflective listening, and more reflecting than asking) than on particular MI techniques.

Bertholet and colleagues (35) found that MI skills measured within three BMI studies were neither robust nor consistent predictors of drinking outcomes. These authors coded audio recordings of 314 BMIs across one US BMI study among middle-aged medical inpatients with unhealthy alcohol use (*N* = 124) and two Swiss BMI studies among young men with binge drinking in a non-clinical setting (*N* = 62 and 128). In all three studies, mean MI counselor’s rating scores were consistent with MI proficiency but most MI skills were not significantly associated with alcohol outcomes at 3/6-month follow-up. In the US study, confrontation (an MI-inconsistent behavior) was associated with more drinking. Limited variability in scores was proposed by the authors to explain this lack of effect.

The limited variability in scores points to methodological limitations of the abovementioned studies. All of these were secondary analyses of the BMI condition of randomized controlled trial, where counselors were trained to perform high-quality BMI. On the other hand, results from meta-analyses cited above [e.g., Ref. (8, 14)] compared BI including MI skills to BI not including this approach. A recent study tried to address these limitations by designing a study including heterogeneous counselors (18 counselors ranging from beginners to MI experts) and comparing participants receiving a BMI with high level of MI skills to those receiving a BMI with low MI skill level and to a control group receiving no BMI (36). This study included non-treatment seeking young men (age 20) screened as hazardous drinkers and found that BMI where MI global ratings (acceptance, empathy, and MI spirit) were high, with no MI-inconsistent behaviors, and with a higher percentage of complex reflections, had better outcomes than those having had no intervention, whereas those with lower scores on these dimensions did not significantly differ from those in the non-intervention control group. Surprisingly, young men receiving BMI with counselors exhibiting a high number of MI-consistent behaviors did not significantly differ in outcome from those in the control group, while those having a lower number of MI-consistent behaviors had significantly better outcomes. The authors proposed that the quality and the exact combination of skills might have mattered more than the quantity.

Two studies by Tollison and colleagues (37, 38) also suggested potential iatrogenic effects of some MI skills. Specifically, these authors examined the association between change in the drinking behavior of the college student and peer facilitator adherence to MI microskills within a BMI. In the first publication (37), results indicated that a higher number of simple reflections were associated with increased rather than decreased drinking at the 3-month assessment among the 67 participants; however, complex reflections were found to attenuate the effects of simple reflections on changes in drinking. In a replication of this study with 327 students (38), higher frequency of both open questions and simple reflections were associated with increases in drinking quantity over 5- and 10-month follow-up. These data are not necessarily in conflict with the view that MI skillfulness is an important component of BI, as greater use of these specific microskills may be indicative of lower overall skill. Together with results from the study by Gaume and colleagues (36), these findings highlight the key importance of competent reflective listening skills (i.e., the use of more complex reflections).

#### Direct advice to reduce or stop drinking, alternative change options, and drinking moderation strategies

In their early review of BI for alcohol problems, Bien and colleagues (14) identified advice as the essence of BI. They further observed that all of the interventions described in their review contained explicit verbal or written advice to reduce or stop drinking. The studies described in their review seldom prescribed a single approach, but advised either a general goal or a range of options. Bien and colleagues (14) consequently posited that this “menu” of alternative change options may increase the likelihood that an individual will find an approach appropriate and acceptable to his or her own situation.

In their meta-analysis of BI for college students, Carey and colleagues (7) showed that face-to-face interventions including moderation strategies were significantly more effective than those not including moderation strategies, in reducing quantity of alcohol consumed, frequency of heavy drinking, and alcohol-related problems. Interventions including alcohol/BAC education also reduced quantity of alcohol consumed significantly more.

On the other hand, in their examination of the components of a BMI for heavy episodic alcohol use among college students, McNally and colleagues (31) showed that students’ (*N* not specified, random half of 73 participants included in the study) subjective report of whether change options had been proposed was not significantly associated with 6-week alcohol outcomes.

In the study by Bertholet and colleagues (35), MI skills measured within three BMI studies were assessed, as previously described, and giving advice was significantly associated with less drinking in one of the studies (BMI with 62 Swiss non-treatment seeking young men with binge drinking in a non-clinical setting).

Meta-analytic findings showed that BI models including advice giving as a strategy had enhanced alcohol outcomes. However, studies that empirically assessed advice giving gave more contrasting results. It should also be noted that this kind of studies was rare (only two studies), and the lack of study of the effects of direct advice is striking.

#### Change plan

Completion of a plan to change alcohol use is an MI component that may represent a culmination of the motivational dialog resulting in verbal statements of intention and a written contract for behavior change (39). Change plans are supposed to be conducted only when the patient is engaged in change, when the client and clinician are working on strengthening commitment to change (“Phase 2” in MI), and if the patient agrees to complete one (40).

Magill and colleagues (39) examined the change plan component within an alcohol-focused BMI among patients included in a hospital-based clinical trial (*N* = 291). This study examined within-session therapist and client language predictors of a client’s decision to complete a written change plan. Logistic regression analyses found that therapist MI-consistent behaviors and client change talk were significant positive predictors, and client sustain talk was a significant negative predictor of the decision to complete a change plan regarding alcohol use. This study provides first elements to link the completion of a change plan with MI-consistent behaviors during a BMI. However, the study did not investigate if the completion of a change plan was associated with follow-up alcohol outcomes.

Lee and colleagues (41) examined the potential predictive role of the quality of an alcohol-related change plan on BMI outcomes within an emergency department sample of injured hazardous drinkers. A mediational analysis framework tested directional hypotheses between pre-treatment readiness, quality of change plan (interventionists completed the change plans with their patients by hand and the quality of the resulting written change plans were coded on 0–3 scale), and treatment outcomes. Participants who completed a BMI and a change plan were included (*N* = 333). Pre-treatment readiness to change was significantly negatively associated with alcohol consequences at 12 months and good-quality change plans. While controlling for pre-treatment readiness to change, good-quality change plan remained a significant predictor of treatment outcomes in the expected direction. Follow-up generalized linear modeling including an interaction term (change plan and pre-treatment readiness) revealed that those with high readiness and a good-quality change plan vs. those with low readiness and a poor-quality change plan had better than predicted outcomes for either readiness or change plan alone. The authors concluded that their findings suggest that the change plan may be an active ingredient of BMI associated with better outcomes over and above the influence of pre-treatment readiness.

If further research and study replication obviously seem necessary, these preliminary elements showed that the completion of a change plan and the quality of this plan might be important components of BMI efficacy. It should be cautioned, however, that change plans will only be completed when sessions have gone well and change has been decided upon, so this evidence may constitute a marker of successful implementation of MI skills resulting in a change plan, rather than suggesting that a change plan may be effective in isolation from other components.

### Mediators

Mediators of treatment effects might be defined as psychological dimensions, psycholinguistic behaviors, or cognitive state that are affected by the intervention and transmitted its effects on targeted behavior change. Effects may be partially or fully mediated in this way. Full-mediation analyses (42) posit how, or by what means, an independent variable (*X*) affects a dependent variable (*Y*) through one or more potential intervening variables, or mediators (*M*). Several paths are tested: path *a* represents the effect of *X* on the proposed mediator(s), path *b* is the effect of *M* on *Y*, and the *ab* path is the indirect effect of *X* on *Y* through *M*. A few BMI studies empirically evaluated full-mediation models (see below). Several studies only investigated either the *a* or *b* paths and are also presented below.

#### Readiness to change

Despite its emphasis on motivation, surprisingly little is known about the role of motivation within BI (and particularly BMI). If motivation or readiness is only thought about and measured pre-intervention, this makes it a moderator rather than only a mediator and such data were not considered here. Motivation, or readiness to change, has been tested as a mediator of BMI’s effects in three studies.

Using data from three published randomized trials implementing BMIs among drinking college students, Borsari and colleagues (43) examined readiness to change as a potential mediator of intervention effects. Two of the three studies indicated that BMI was associated with increases in motivation to change alcohol use that are apparent immediately after BMI sessions and persist up to 6-month post-intervention. However, readiness to change did not appear to be a mechanism of behavior change, as it did not mediate reductions in alcohol use or problems in any of the studies.

Barnett and colleagues (44) evaluated several moderators and mediators of alcohol BMI for young adults (18–24 years; *N* = 172) conducted in an emergency department. Readiness to change was evaluated as a mediator of the intervention’s efficacy but no significant mediation was found. BMI was associated at the trend level to higher readiness to change post-intervention (*p* < 0.1), but higher readiness to change did not predict better alcohol outcome.

Stein and colleagues (45) examined readiness to change drinking as a mediator of the effects of BMI on alcohol-related consequences also within an emergency department setting. Participants were randomized into three conditions: (a) standard care plus assessment, (b) standard care plus BMI, and (c) standard care plus BMI plus a booster session. Patients receiving any BMI maintained higher readiness scores 3 months after treatment than did patients receiving standard care. At 12-month follow-up, BMI plus a booster session patients had significantly reduced alcohol consequences more than standard-care patients. However, readiness mediated treatment effects only for those highly motivated to change prior to the intervention but not for those with low pre-intervention motivation. Authors speculated that two sessions of BMI will be sufficient to sustain the motivation to change for those more highly motivated to change prior to the intervention, but for those less ready to change prior to the intervention, two sessions of BMI are insufficient to motivate the patient to mobilize his or her resources to initiate or sustain the targeted behavioral change.

Even if motivation and readiness to change are theoretically central constructs of all BIs (and not just BMI), there are sparse and unsupportive data as mediators of BMI effects. There are also few investigations of motivation within the alcohol treatment literature [see Ref. (46)]. Difficulties in measurement may explain these findings of lack of effect. Interestingly, more detailed analyses, such as those proposed by Stein and colleagues (45) might help understand how interventions work. Using a moderated mediation framework, these authors showed that readiness to change did mediate BMI effects only under specific circumstances. Such conditional effects might help understand inconsistent findings.

#### Change talk

Motivational interviewing has been described as a collaborative conversation style for strengthening a person’s own motivation and commitment to change (30) and central to it is the hypothesis that people are more likely to be persuaded by what they hear themselves say (30, 47). Client statements toward and against change (or change talk and sustain talk) are thus hypothesized to mediate MI intervention efficacy (48).

Baer and colleagues (49) analyzed 54 recordings of BMI with homeless adolescents, who used alcohol or illicit substances but were not seeking treatment. Results indicated that statements about desire not to change or inability to change, although infrequent (mean = 0.61/5 min), were strongly predictive of less abstinence of alcohol and substance use at both 1- and 3-month follow-up. Statements about reasons for change were associated with greater reductions in days of substance use at 1-month assessment. Commitment language was not associated with outcomes.

Gaume and colleagues (33, 50) and Bertholet and colleagues (51) assessed change talk during 97 BMIs in an emergency department. They showed that MI-consistent behaviors were the only counselor behaviors that were significantly more likely to be followed by patient change talk overall (i.e., aggregating the different sub-dimensions such as ability, desire, commitment to change, etc.) (50). Using the same data, these authors showed that patient ability to change expressed during BMI was a significant predictor of alcohol use at 12-month follow-up (33). Patient change talk overall was not tested as a predictor of alcohol outcomes so that a complete chain from counselor’s behaviors to patient change talk to outcome cannot be derived from these two studies. Nevertheless, another analysis using these data (51) suggested that change talk might have been a mechanism of change within this intervention. Using a hidden Markov model, analyses showed that a patient’s attitude “toward change” at the end of the intervention was associated with improved outcomes at follow-up, independent of the type of change talk at the beginning of the intervention.

Similar analyses were carried by the same group using data on BMI among young men from the general population (52, 53). Again, MI-consistent behaviors were the only counselor behaviors that were significantly more likely to be followed by patient change talk overall (53) and alcohol use at 6-month follow-up was significantly predicted by a change talk variable combining ability, desire, and need to change or not to change (52). Patient change talk overall was not a significant predictor of alcohol outcomes but change talk averaged strength (i.e., a composite variable combining statements expressed toward change and away from change) trended toward prediction of alcohol outcome (*p* = 0.08). Again, the complete chain from counselor’s behaviors to patient change talk to outcome was not observed in these two studies, leaving the mediation hypothesis needing to be further tested.

A full-mediation analysis was addressed in the paper by Vader and colleagues (54). In this study, the authors examined the relationship between counselor behaviors and client change talk, personalized feedback and change talk, and client change talk and client drinking outcome (composite score consisting of drinks per week, peak blood alcohol concentration, and protective drinking strategies), in a sample of heavy-drinking college students. MI was delivered in a single session with or without a personalized feedback report. A. In the MI with feedback group, MI-consistent counselor’s behaviors were positively associated with client change talk. After receiving feedback, MI with feedback clients showed lower levels of sustain talk, relative to MI only clients. Finally, in the MI with feedback group, clients with greater change talk showed improved drinking outcomes at 3 months, while clients with greater sustain talk showed poorer drinking outcomes. Building on these positive findings within the MI with feedback group, the authors tested change talk as a mediator between MI-consistent behaviors and drinking outcomes but observed a non-significant indirect effect (i.e., no evidence of mediation).

#### Self-efficacy

Enhancing client self-efficacy was previously a central component of MI (40), which has more recently been referred to as strengthening confidence to change (30). Research on self-efficacy as a mediator has shown mixed findings, but self-efficacy has not been well evaluated in studies of BMI for alcohol use (44).

Among the heavy-drinking college students who were randomly assigned to a BMI (*N* not specified, random half of 73 participants included in the study) in the study by McNally and colleagues (31), the participants’ subjective experience of how much the counselor encouraged self-efficacy was not significantly associated with 6-week alcohol outcomes.

Barnett and colleagues (44) evaluated moderators and mediators of brief alcohol interventions conducted in an emergency department. Patients (18–24 years; *N* = 172) received a BMI with personalized feedback or feedback only, with 1- and 3-month booster sessions and 6- and 12-month follow-up. Among the tested mediators, self-efficacy was not significant. Individual path analysis showed that higher self-efficacy was significantly associated with lower levels of alcohol use, but randomization status (BMI vs. feedback only) was not related to a shift in self-efficacy.

#### Enhancement of discrepancy

Motivational interviewing seeks to develop and resolve discrepancy between the individual’s current behavior and broader life goals and values (30). McNally and colleagues (31) examined the effects of a BMI for heavy, episodic alcohol use on discrepancy-related psychological processes. Heavy-drinking college students (*N* = 73) were randomly assigned to a BMI or an assessment-only control condition. Cognitive (actual–ideal discrepancy) and affective (cognitive dissonance) discrepancy processes were assessed at baseline and immediately following the experimental manipulation. At 6-week follow-up, BMI participants demonstrated significantly greater reductions in problematic drinking than controls. Moreover, actual–ideal discrepancy and negative, self-focused dissonance were significantly increased following the intervention (discomfort-related dissonance was not) and were correlated with the outcome alcohol involvement. These discrepancy processes did not, however, significantly mediate the relationship between condition and outcome.

Within the same study (31), the authors also tested whether MI components assessed after the BMI (*N* not specified, random half of the 73 participants) were related with alcohol outcome at 6-week follow-up (controlling for baseline drinking). The findings suggested that BMI participants who reported enhanced awareness of their drinking were more likely to show better outcomes. In their discussion, these authors suggested that this raised awareness might be conceptualized as having a direct relationship to discrepancy-related psychological processes as students’ conscious awareness of their actual drinking patterns (enhanced through personalized feedback and/or through the MI format discussion) might raise their cognitive or affective discrepancy.

In their study evaluating moderators and mediators of emergency department based BMI for young adults (18–24 years; *n* = 172), Barnett and colleagues (44) tested a risk-benefit difference score as a potential mediator of the effect of BMI. Analysis of individual paths showed that as compared to feedback only, the BMI with personalized feedback group did not show the expected shift in perceived risks/benefits of drinking at 6-month follow-up. On the other hand, a shift in perceived risks/benefits at 6-month follow-up showed a trend toward lower alcohol use (*p* < 0.1) at 12-month follow-up. No significant mediation was observed.

#### Norm perceptions

In their trial on BMI with or without feedback to reduce heavy drinking among college students, Walters and colleagues (22) also tested if norm perceptions did mediate the effect of the intervention. They found that (a) BMI with feedback (*N* = 73) significantly affected the alcohol outcomes as compared to the assessment-only control condition (*N* = 69); (b) the intervention reduced norm discrepancies at 6 months, becoming more accurate in their norm estimates (i.e., smaller discrepancies); (c) smaller norm discrepancies were associated with better alcohol outcomes; and (d) adjusting for norm discrepancies reduced the magnitude of the intervention effect on alcohol outcomes.

#### Use of protective behavior and alcohol treatment seeking

Two other mediators were tested in two papers already presented above, but were not significant.

In their trial of BMI with or without feedback to reduce heavy drinking among college students, Walters and colleagues (22) also tested if the use of protective behaviors when drinking alcohol [e.g., set a target for number of drinks, alternate alcoholic and non-alcoholic drinks, and use a designated driver; (55)] mediated the effect of the BMI with feedback (*N* = 73) as compared to the assessment-only control condition (*N* = 69). They indicated that protective behaviors were only weakly related to the intervention and to the 6-month outcomes (no statistics reported) and did not mediate the intervention effect.

Alcohol treatment seeking was tested by Barnett and colleagues (44) within a BMI with personalized feedback for young adults (18–24 years; *n* = 172). Analysis of individual paths showed that as compared to feedback only, the BMI group showed a trend toward greater treatment seeking at 6-month follow-up (*p* < 0.1), but treatment seeking at 6 months was not significantly related with lower alcohol use at 12-month follow-up.

## Discussion

We conducted a review of studies reporting mechanisms of action of BI for hazardous or harmful alcohol use. Overall, BI active ingredients have been scarcely investigated, almost only within BMI studies, and mostly among patients in the emergency room, non-treatment seeking young adults, and US college student populations. This is surprising considering that BI evidence of efficacy comes mostly and primarily from studies conducted in primary health care settings. It may indicate that null trials have led researchers to investigate the BI black box in search for clues as to which elements of BI may carry efficacy, a task they somewhat did not carry in the context of efficacious studies. As such, it should be noted that some of the evidence summarized herein comes from null trials and that almost all of it comes from research conducted in settings in which evidence of BI efficacy should be considered inconclusive [with the exception of the US colleges, see Ref. (7, 8)].

On the basis of the evidence reviewed herein, we summarize that:

(1) personalized feedback may be an effective component;
(2) decisional balance showed mixed findings, which tend to suggest a potential detrimental effect;
(3) some MI skills and techniques showed mixed findings;
(4) direct advice to reduce or stop drinking has not been empirically studied; presenting alternative change options, and relatedly using a range of moderation strategies are promising but need further study;
(5) change plan exercises are promising and need to be further studied as discrete components and also in relation to MI skills;
(6) client change talk is a potential mediator of BMI effects;
(7) change in norm perceptions and enhanced discrepancy have received preliminary support, but from only one study each;
(8) enhanced awareness of drinking, perceived risks/benefits of alcohol use, alcohol treatment seeking, and self-efficacy have as yet found no significant support as mediators, but were seldom studied; and
(9) readiness to change was only partially supported as a mediator of BI effect.

Readers familiar with the BI literature will notice that the conclusions summarized here include active ingredients from different models of BI (e.g., normative feedback, MI, and psycho-education). The paucity of studies, especially of studies designed specifically to investigate active ingredients of BI shows that more research is needed. In addition, most of the evidence on active ingredients comes from studies conducted on one particular subtype of BIs, i.e., those derived or adapted from MI, and is limited to particular settings and populations (college students and young adults, emergency department). It is important that active ingredients can be identified in settings in which BI has been shown efficacious, like primary health care (1). For now, it is still unknown how BI achieves the effects observed in these randomized trials.

Another important area for future research is BI effects on the moderators, i.e., for whom or under which conditions BI is effective (or not). These were not the focus of our study (here we have investigated how BI works rather than for whom), and we suggest a contribution to be made on studying moderators effects, but also on investigating moderators of mediators effects. Determining what are the active ingredients of BI, and whether these ingredients are robust across settings and populations, is crucial to further develop effective interventions and will aid understanding of observed discrepancies between studies on both mediators and effects. Which combination (if any) of active ingredients (possibly across the different theoretical models of BI) is most effective deserves to be investigated but must await progress in the areas identified for further study.

## Conflict of Interest Statement

No conflicts of interest are to be reported. The authors disclose that part of the studies reviewed in the present article were their own work. They tried to consider those as objectively as possible, but some partiality might have remained.
